# Disseminated Tuberculosis Presenting With a Nodo-Colonic Fistula in a Newly Diagnosed HIV-Positive Patient: A Case Report From Tanzania

**DOI:** 10.7759/cureus.106369

**Published:** 2026-04-03

**Authors:** Samina Somji, Salim Surani, Joshua Ngimbwa, Tevin Kahwa, Frank Swai, Fatema Tayabali, Bishoy Rofaeil

**Affiliations:** 1 Internal Medicine, Medical College, Aga Khan University, Dar es Salaam, TZA; 2 Medicine, Valley Coastal Bend Veterans Administration, Corpus Christi, USA; 3 Medicine, University of Houston, Houston, USA; 4 Medicine, Aga Khan University, Nairobi, KEN; 5 Anesthesiology, Mayo Clinic, Rochester, USA

**Keywords:** diarrhea, disseminated tuberculosis, hiv, human immunodeficiency virus, nodo-colonic fistula, tuberculosis

## Abstract

We present the case of a 35-year-old male with no known comorbidities who presented with chronic gastrointestinal (GI) and constitutional symptoms and was empirically treated with several antibiotics during multiple clinic visits at different health facilities. The laboratory test sent included a stool GeneXpert, which was positive for Mycobacterium tuberculosis (MTB). CT imaging revealed multiple necrotic abdominal and retroperitoneal lymph nodes, hypoenhancing splenic lesions, a nodo-colonic fistula between the necrotic retroperitoneal lymph nodes and the adjacent colon, and a mild left-sided pleural effusion. Colonoscopy demonstrated caseating discharge from the fistulous tract 40 cm from the anal verge, and histology showed abundant neutrophils and histiocytes with areas of caseous-like necrosis. The patient was diagnosed with disseminated tuberculosis (TB) with newly diagnosed HIV stage 4. Antituberculous therapy was initiated, followed by antiretroviral therapy (ART) two weeks later in accordance with World Health Organization and national treatment guidelines.

This case highlights the need for a thorough evaluation of a patient with a history of chronic GI symptoms and the importance of maintaining a high index of suspicion when diagnosing disseminated TB. In TB-endemic regions, a differential diagnosis of TB should be sought in patients presenting with GI and constitutional symptoms, and such symptoms should prompt an HIV test. Imaging plays a pivotal role in identifying key features such as lymphadenopathy and fistulous tracts, and colonoscopy can show a caseating discharge, which should raise suspicion for chronic infectious or granulomatous diseases, including TB. Although rare, nodo-colonic fistula formation can occur as a complication of abdominal TB. Conservative management can be effective in stable patients, potentially avoiding the morbidity of surgical intervention, as demonstrated in this case. Comprehensive management, including prompt initiation of anti-TB therapy, timely ART, prophylaxis for opportunistic infections, and nutritional support, is crucial to optimize outcomes in such complex presentations.

## Introduction

Tuberculosis (TB) remains a major global health concern, particularly in Tanzania, a country with a high TB burden and substantial TB/HIV coinfection. In HIV-infected populations, TB is the leading cause of death [1]. While pulmonary disease predominates, extrapulmonary tuberculosis (EPTB) contributes to a significant proportion of cases (~20% in Tanzania), and within EPTB, abdominal TB accounts for an estimated 11-16% [2].

Disseminated TB is a severe form of Mycobacterium tuberculosis (MTB) infection resulting from lymphohematogenous spread, with involvement of two or more noncontiguous organ systems. It occurs most commonly in individuals with advanced immunosuppression, particularly those living with HIV, where impaired cell-mediated immunity leads to atypical and severe disease [3,4]. Clinical manifestations are often nonspecific, including constitutional symptoms and multisystem involvement such as lymphadenopathy, splenic or pleural disease, and gastrointestinal (GI) involvement, frequently mimicking malignancy or other chronic inflammatory conditions [3,4]. In TB-endemic settings such as Tanzania, delayed diagnosis is common and contributes to increased morbidity and mortality, underscoring the importance of early recognition, imaging-based evaluation, and prompt initiation of antituberculous therapy with timely antiretroviral treatment.

In sub-Saharan Africa, including Tanzania, disseminated TB remains an important diagnostic challenge among adults presenting late or having underlying immunosuppression, including HIV infection [5,6]. It may present with simultaneous pulmonary involvement, abdominal TB, and rare complications such as entero- or colocutaneous fistulas [2].

Clinically, abdominal TB often presents with chronic GI and constitutional symptoms and is associated with diagnostic delays. In a northern Tanzanian cohort of 256 patients, nearly half presented with intestinal obstruction and 41% with peritonitis; fistulae were uncommon (2.3%) but were documented [7].

A nodo-colonic fistula due to TB is an extremely rare manifestation of abdominal TB, with no well-defined prevalence in the literature because most evidence comes from isolated case reports and small case series rather than large epidemiological studies [1,7]. We report this case to highlight an uncommon manifestation of disseminated TB. Early recognition is crucial for clinicians managing patients with chronic GI symptoms in TB-endemic regions like Tanzania. Documenting such presentations enhances awareness of atypical TB manifestations and reinforces the importance of comprehensive evaluation, including imaging, histopathology, and HIV testing, to enable timely diagnosis and appropriate management.

## Case presentation

A 35-year-old male with no known comorbidities presented with a two-month history of nonprojectile postprandial vomiting of food contents and brown, loose, watery stools occurring two to three hours after meals. He also reported constitutional symptoms of a two-month duration, including easy fatigability, unintentional weight loss of 5 kg, intermittent subjective high-grade fevers and night sweats, and dull, poorly localized abdominal pain. He denied any history of cough, chest pain, hemoptysis, dyspnea, altered mentation, or previous history of TB. He was treated at multiple health facilities for gastroenteritis with several antibiotics; the names and duration of antibiotics he could not recall, but he said he had completed the courses as prescribed with no relief of his symptoms. There was no prior history of surgery, blood transfusions, or allergies.

On general examination, the patient was alert and afebrile but appeared mildly pale and jaundiced. His vital signs were stable, and systemic examination was unremarkable. He was admitted for further evaluation of chronic diarrhea and vomiting with constitutional symptoms.

Initial laboratory investigations revealed microcytic anemia with evidence of hemolysis. The peripheral smear demonstrated marked anisopoikilocytosis with schistocytes, spherocytes, and microcytosis. These findings suggested a mixed picture involving iron deficiency (or anemia of chronic disease) and a hemolytic process, possibly related to hypersplenism. White blood cell morphology showed lymphopenia with mature segmented forms, and platelet count was within normal limits, as seen in Table 1 below.

**Table 1:** Laboratory results of the patient. ESR, erythrocyte sedimentation rate; AST, aspartate aminotransferase; ALT, alanine aminotransferase; ALP, alkaline phosphatase; GGT, gamma-glutamyl transferase; LAM, lipoarabinomannan; ADA, adenosine deaminase; HBsAg, hepatitis B surface antigen; TB, tuberculosis

Test	Result	Reference Range	Units
ESR	128	0-20 (adult male)/0-30 (adult female)	mm/hr
Hemoglobin	7.9	13.0-17.0 (male)/12.0-15.0 (female)	g/dL
White Blood Cells	9.43	4.0-11.0	×10^9^/L
Platelets	411	150-400	×10^9^/L
AST	35	10-40	U/L
ALT	43	7-56	U/L
ALP	234	44-147	U/L
GGT	249	9-48	U/L
Total Bilirubin	5	0.2-1.2	mg/dL
Direct Bilirubin	4.98	0.0-0.3	mg/dL
Creatinine	51	62-115 (male)/53-97 (female)	µmol/L
Hepatitis C Antibodies	Negative	Negative	-
HBsAg	Negative	Negative	-
Sputum and Stool GeneXpert (TB)	Detected (positive)	Not detected	-
Urinary LAM	Positive	Negative	-
Serum ADA	14	0-30	U/L
CD4 Count	14	500-1500	cells/µL
Syphilis Serology	Negative	Negative	-

**Figure 1:**
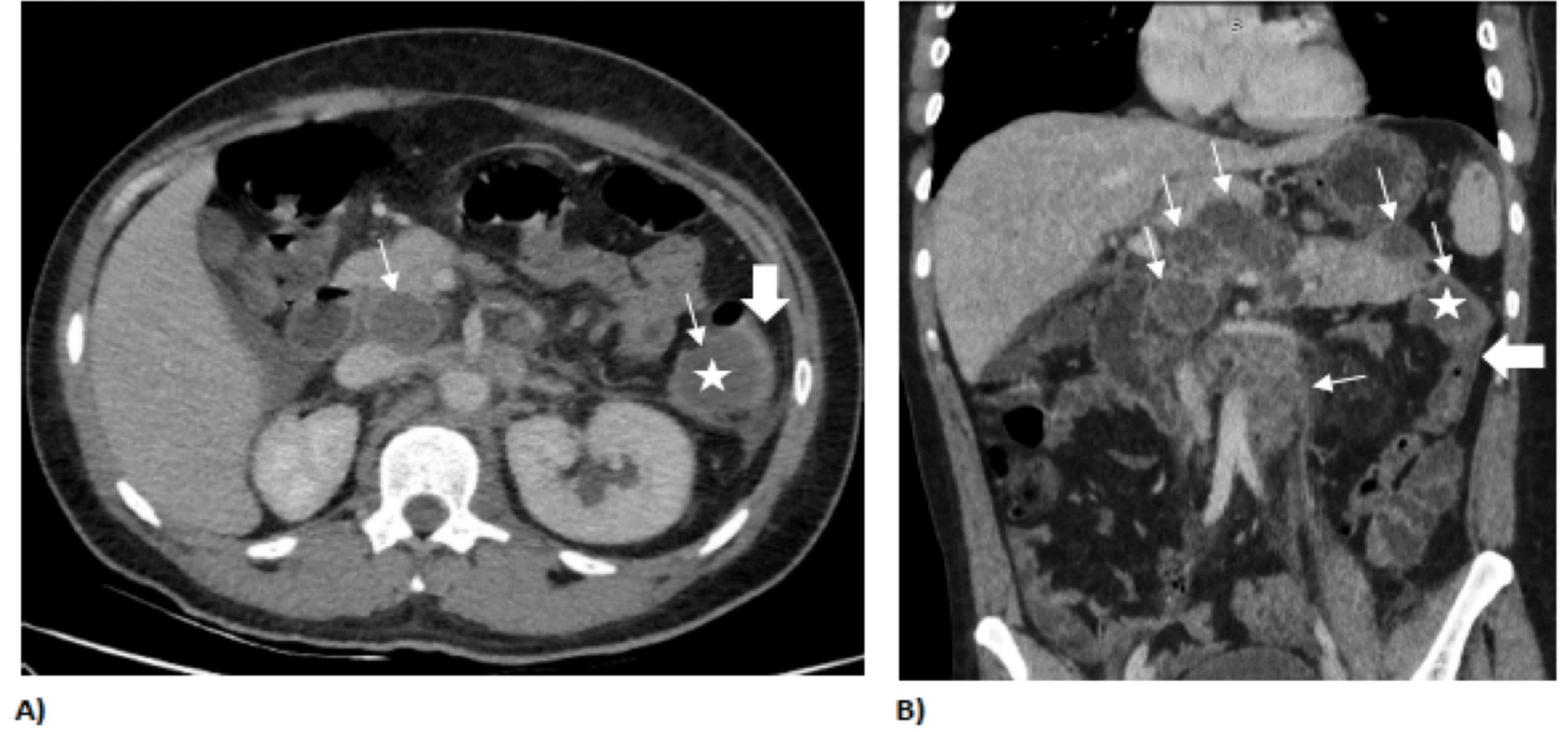
Multiple necrotic abdominal and retroperitoneal lymphadenopathy on abdominal CT with IV contrast. Axial (A) and coronal (B) study before IV contrast, before colonoscopy, showing multiple retroperitoneal necrotic lymphadenopathy (thin white arrows), with one of them (asterisk) intimately related to the most proximal descending colon (thick white arrow). IV, intravenous

An abdominal ultrasound showed an epigastric mass with mild intra-abdominal ascites. An intravenous (IV) contrast-enhanced CT scan of the abdomen revealed extensive necrotic abdominal and retroperitoneal lymphadenopathy, with the largest measuring 6×5.5 cm, multiple hypoenhancing splenic lesions, and a mild left-sided pleural effusion, as shown in Figure 1.

**Figure 2:**
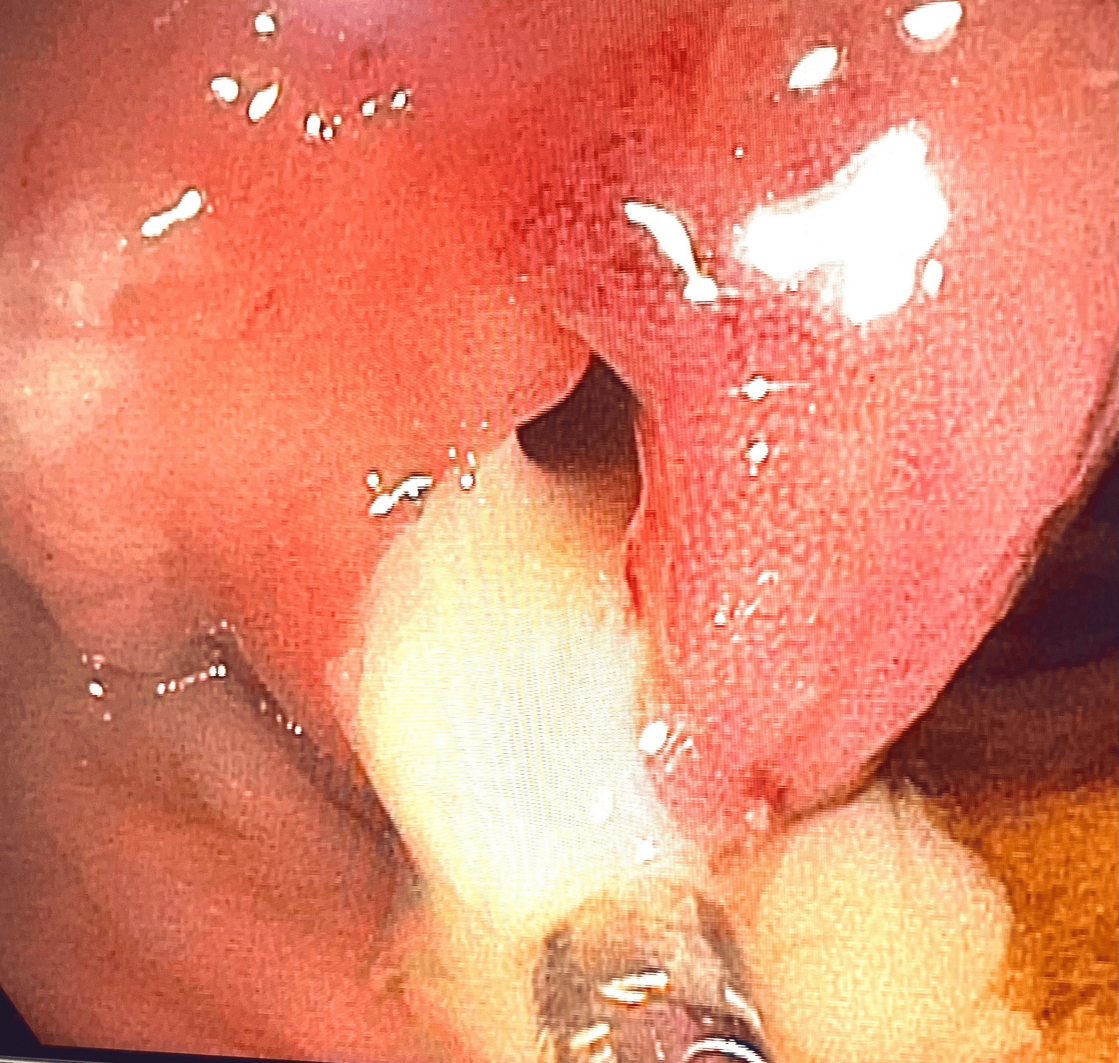
Colonoscopy revealing a protruding orifice observed approximately 40 cm from the anal verge, discharging white, caseous material.

Given the GI symptoms and the abdominal findings, a colonoscopy was performed and revealed a bulging orifice at 40 cm from the anal verge, draining white caseating material, consistent with intestinal TB, as shown in Figure 2. The caseating fluid was fully drained during the procedure.

**Figure 3:**
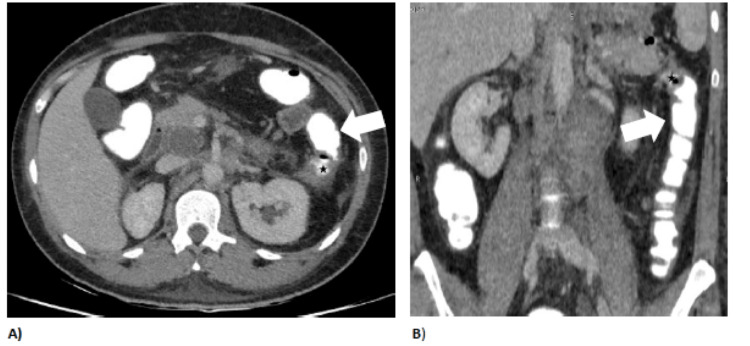
CT with IV and rectal contrast after colonoscopy and before initiation of treatment. Axial (A) and coronal (B) CT abdomen study with IV contrast after colonoscopy showing significant volume reduction and evacuation with air-fluid level of the necrotic lymphadenopathy (asterisk) seen intimately related to the most proximal descending colon (thick white arrow). The rest of the necrotic lymphadenopathy are unchanged. IV, intravenous

Postcolonoscopy, a CT scan of the whole abdomen with rectal contrast was performed to further evaluate the nature of the fistula, which revealed the presence of a nodo-colonic fistula between the necrotic retroperitoneal lymph nodes and the adjacent colon, evidenced by air-fluid levels and regression in lymph node size due to draining performed during the colonoscopy, as shown in Figure 3.

Microbiological testing supported the diagnosis of TB. GeneXpert testing from the stool specimen and sputum detected MTB at trace levels, with rifampicin resistance indeterminate, likely due to the low bacterial load. These findings confirmed active pulmonary and EPTB infection. Acid-fast bacilli and sputum culture for TB were not attempted. Further evaluation confirmed that the patient was HIV-positive with an HIV-1 viral load of 699,000 copies/mL and a CD4 count of 14 cells/μL (3.08%). This significantly increased his risk for opportunistic infections and immune reconstitution inflammatory syndrome (IRIS). Urinary lipoarabinomannan (LAM) assay was positive, supporting the diagnosis of disseminated TB in the context of advanced immunosuppression (CD4 count: 14 cells/μL).

The final diagnosis of HIV stage 4 with disseminated TB was made, and the patient was initiated on first-line anti-TB therapy comprising rifampicin (150 mg), isoniazid (75 mg), pyrazinamide (400 mg), and ethambutol (275 mg), with a dosing regimen of four tablets once daily. This follows the Tanzania TB treatment guidelines, whereby all four medications are given for two months and then only isoniazid and rifampicin are given for four months to make a total of six months treatment duration. In addition, due to signs of nutritional compromise, the patient was started on an adequate nutritional supplement. He was prescribed folic acid 5 mg daily, cotrimoxazole 960 mg once daily for opportunistic infection prophylaxis, given his profound immunosuppression, as well as pyridoxine (vitamin B6) 25 mg daily as prophylaxis for peripheral neuropathy since he was initiated on anti-TB treatment with isoniazid. The nodo-colonic fistula was managed conservatively. As per WHO and national guidelines, antiretroviral therapy (ART) was initiated two weeks after starting anti-TB treatment with a regimen of tenofovir (300 mg), lamivudine (150 mg), and dolutegravir adjusted to 50 mg twice daily due to rifampicin interaction.

The patient continued to do well at follow-up visits with resolution of his symptoms, and he gained 8 kg over four months. A repeat CT done at four months showed resolution of lymph nodes, and colonoscopy also revealed resolution of caseating discharge and reduction in the size of the fistula.

## Discussion

Our patient presented with chronic GI and constitutional complaints, which may signify intestinal involvement of TB but are also easily misattributed to other chronic GI disorders, highlighting the diagnostic difficulty in such settings.

In advanced HIV infection, several opportunistic infections and malignancies may present with overlapping radiologic features, including disseminated Mycobacterium avium complex (MAC), histoplasmosis, cryptococcosis, talaromycosis (Talaromyces marneffei), and HIV-associated lymphoma. Disseminated MAC typically presents with diffuse lymphadenopathy, hepatosplenomegaly, and constitutional symptoms, often with minimal pulmonary cavitation. Histoplasmosis and talaromycosis may mimic TB with reticulonodular infiltrates and systemic dissemination, particularly in endemic regions, while cryptococcosis may involve pulmonary nodules or masses with concomitant central nervous system disease. HIV-associated lymphoma should also be considered, particularly in the presence of bulky lymphadenopathy, extranodal involvement, or poor response to antimicrobial therapy. In our case, the presence of microbiologically confirmed MTB, together with characteristic clinical and radiologic findings and subsequent response to antituberculous therapy, supported TB as the unifying diagnosis.

HIV coinfection plays a pivotal role in the development of disseminated and atypical TB presentations. In Tanzania, approximately 17-20% of TB patients are HIV-positive, and immunosuppression markedly increases the likelihood of disseminated disease [8]. Our patient’s profoundly low CD4 count of 14 cells/µL explains the unusual presentation and the severity of disease, as HIV weakens granuloma formation and impairs immune control of mycobacterial proliferation.

A particularly rare finding in this case was the nodo-colonic fistula, which was demonstrated radiologically. Fistula formation in abdominal TB is uncommon, with enteroenteric and enterovesical fistulae more frequently described [9]. Involvement of the colon by contiguous spread from necrotic lymph nodes is an unusual presentation but has been reported in a few cases. The mechanism is thought to involve caseating necrosis within abdominal lymph nodes, leading to liquefaction and eventual erosion into adjacent bowel loops, which was observed in our patient [9]. Interestingly, while some nodo-colonic fistulae appear paradoxically after initiation of anti-TB therapy [9], in our patient, however, the fistula was already present at the time of diagnosis, likely reflecting late presentation in the context of untreated HIV infection.

Differential diagnoses for a nodo-colic fistula in advanced HIV include tuberculous lymphadenitis with secondary bowel erosion, HIV-associated lymphoma with direct bowel infiltration, Crohn’s disease, disseminated fungal infections such as histoplasmosis, and abdominal actinomycosis [2]. TB commonly causes caseating lymphadenitis with contiguous spread to adjacent bowel, predisposing to fistula formation. In contrast, lymphoma typically presents with bulky lymphadenopathy and mass effect with bowel infiltration, while Crohn’s disease is characterized by transmural inflammation and skip lesions leading to fistulization. Disseminated fungal infections may mimic TB with nodal and GI involvement, particularly in immunocompromised hosts, whereas actinomycosis is associated with chronic suppurative inflammation and sinus tract formation. In our case, microbiological confirmation of MTB, along with compatible clinical and radiologic findings and response to antituberculous therapy, established TB as the underlying cause.

The diagnosis of disseminated TB requires a high index of suspicion because clinical manifestations are nonspecific and overlap with malignancy and other chronic infections. Imaging with ultrasound and CT in our case demonstrated classic findings of necrotic lymphadenopathy with rim enhancement and splenic lesions with mild pleural effusion, which are strongly suggestive of disseminated TB in endemic regions [2]. Colonoscopy contributed by providing direct evidence of caseating discharge, while microbiological confirmation came from GeneXpert, which detected MTB in both stool and sputum specimens. Although stool GeneXpert has reduced sensitivity compared with tissue biopsy, it is an increasingly valuable noninvasive diagnostic tool in resource-limited settings, particularly in disseminated TB [10]. The detection of MTB in sputum, despite no respiratory symptoms, confirmed pulmonary involvement, underscoring the systemic spread of disease.

Management of disseminated TB relies primarily on anti-TB chemotherapy. Our patient was initiated on standard first-line treatment with rifampicin, isoniazid, pyrazinamide, and ethambutol, which remains the cornerstone of care [11]. Despite the presence of a nodo-colonic fistula, conservative treatment was favored, and follow-up imaging confirmed regression in lymph node size and the fistula. This approach is supported by reports indicating that many TB-related fistulae heal spontaneously under effective medical therapy, with surgery reserved for persistent or complicated cases [11]. Nutritional rehabilitation was also prioritized in our patient, as malnutrition is both a cause and consequence of disseminated TB. Supplementation with high-protein formulas and folic acid supported recovery, while cotrimoxazole prophylaxis was appropriately initiated to prevent opportunistic infections in the setting of severe immunosuppression.

The prognosis of disseminated TB in advanced HIV remains guarded. Mortality is significantly higher among HIV-infected patients, particularly those with CD4 counts under 50 cells/µL [11]. While fistula closure is anticipated with chemotherapy, persistence or recurrence may necessitate surgical intervention. Our patient continues to be followed closely to monitor treatment response, nutritional recovery, and potential complications.

## Conclusions

This case highlights several important lessons for clinical practice. TB should remain a key differential diagnosis in patients presenting with chronic GI and constitutional symptoms in endemic settings and should prompt HIV testing. Disseminated TB in advanced HIV may present with rare complications such as nodo-colonic fistulae due to necrotic lymphadenitis eroding into adjacent bowel, requiring a high index of suspicion. Stool GeneXpert may serve as a valuable diagnostic adjunct in resource-limited settings where conventional sampling is challenging. In selected stable patients, conservative management with antituberculous therapy alone may be effective for TB-related fistulae, potentially avoiding surgical morbidity. Finally, comprehensive care, including early initiation of ART, appropriate opportunistic infection prophylaxis, and nutritional rehabilitation, is essential to optimize outcomes in such complex presentations.
